# Supplementary material for: Glycan reactive anti-HIV-1 antibodies bind the SARS-CoV-2 spike protein but do not block viral entry
Source: Sci Rep. 2021 Jun 14;11:12448. doi: 10.1038/s41598-021-91746-7 (PMC8203731; doi:10.1038/s41598-021-91746-7)
Supplement: Supplementary file 1 — Supplementary Information. [file 41598_2021_91746_MOESM1_ESM.pdf]

Supplementary Information (Table S1 and Figures S1-8)

Glycan reactive anti-HIV-1 antibodies bind the SARS-CoV-2 spike protein but do not block viral entry

Dhiraj Mannar, Karoline Leopold, and Sriram Subramaniam

Department of Biochemistry and Molecular Biology,

University of British Columbia, Vancouver, British Columbia, Canada

Correspondence: sriram.subramaniam@ubc.ca

Table S1. HIV gp120 epitopes recognized by antibodies selected for SARS-CoV-2 S cross reactivity screening, along with antibody IgG isotypes.

Antibody	gp120 epitope	IgG isotype	Reference
2G12	N-linked glycans in the C2, C3, V4, and C4 domains	IgG_1 κ	[1]
PGT121	N332-centered oligomannose patch on the V3 loop, GDIR motif in V3 loop	IgG_1 λ	[2–4]
PGT126	N332-centered oligomannose patch of the V3 loop, GDIR motif in V3 loop	IgG_1 λ	[2–4]
PGT128	V3-glycan, GDIR motif in V3 loop	IgG_1 λ	[2,5,6]
PGT145	V1-V2 Glycans, residues within C-strand of V1/V2 and the C1 region near the base of V1	IgG_1 κ	[2,7]
PG9	V1-V2 Glycans, residues within C strand of V1/V2	IgG_1 λ	[8,9]
PG16	V1-V2 Glycans, residues within V1–V2	IgG_1 λ	[8,10,11]

10-1074	V3-glycan, N332 glycosylation dependent, GDIR motif in V3 loop	IgG_1 λ	[12,13]
35O22	Glycan and peptides at the gp120–gp41 interface	IgG_1	[14]
VRC01	CD4-binding site	IgG_1 κ	[15]
VRC03	CD4-binding site	IgG_1 κ	[15]

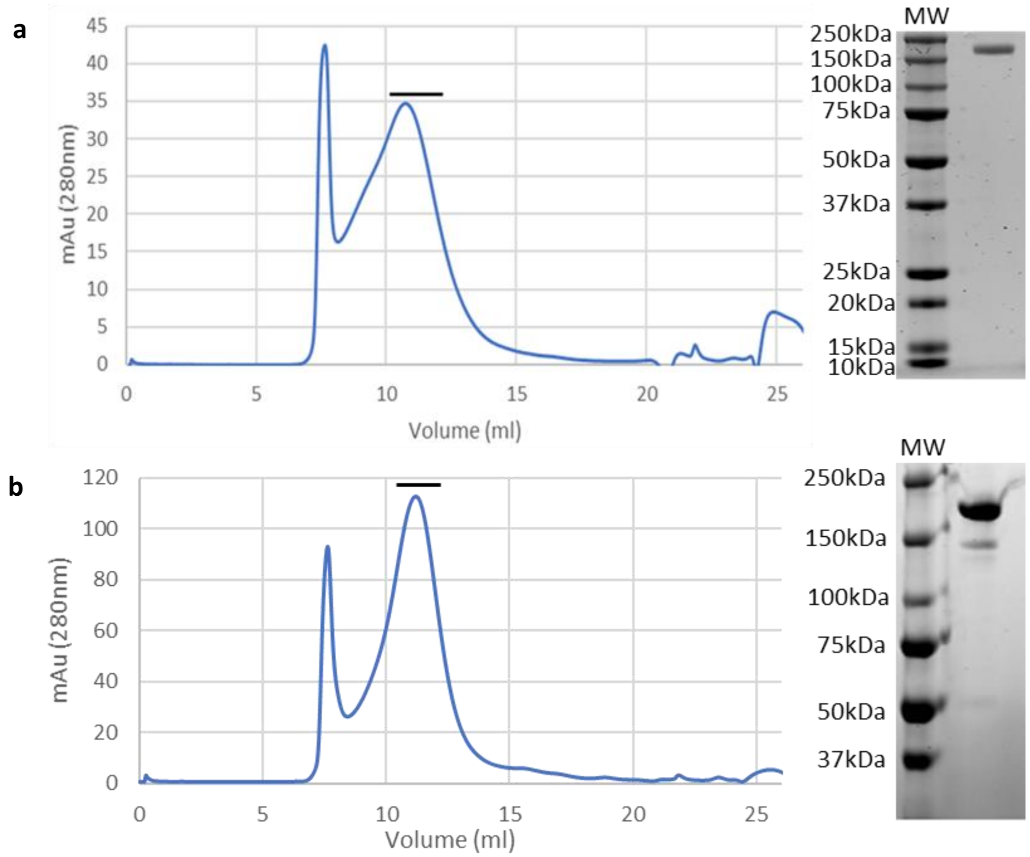

Figure S1. Purification of the SARS-CoV-2 S ectodomain expressed in cells either in the presence or absence of kifunensine. (a,b) Size exclusion chromatography profile of affinity purified SARS-CoV-2 S ectodomain expressed in cells either in the absence **(a)** or presence **(b)** of kifunensine run on a Superose 6 10/30 column, along with SDS-PAGE analyses of pooled and concentrated SARS-CoV-2 S ectodomain. The black line indicates fractions retrieved and pooled for SDS-PAGE analysis. Gels were stained with Coomassie. (MW: molecular weight ladder). See figure S6 in supplementary information for uncropped gel images.

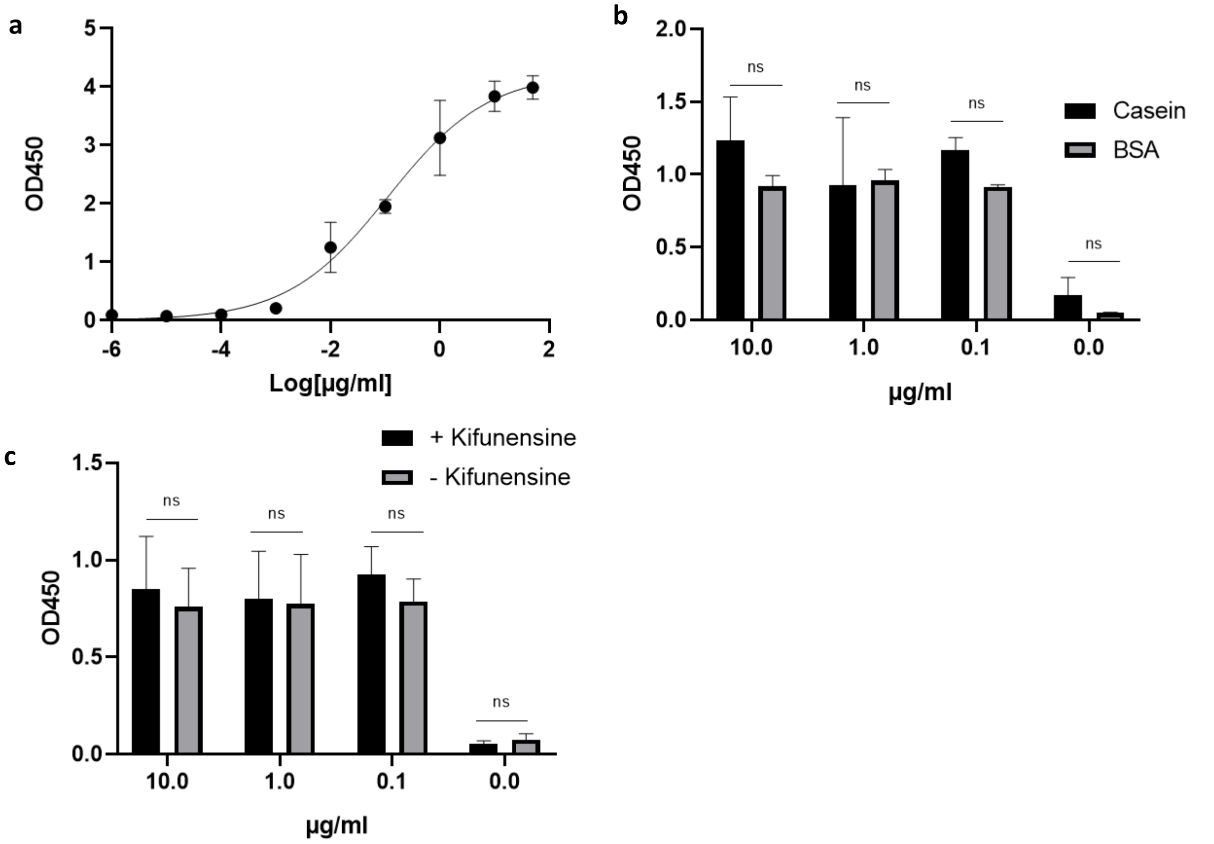

Figure S2. Analysis of VH-Fc ab8 – SARS-CoV-2 spike ectodomain interactions via ELISA. (a) SARS-CoV-2 S binding by VH-FC ab8, carried out in casein-based buffer. **(b)** SARS-CoV-2 S binding by VH-FC ab8 in the presence of BSA or casein-based buffers. **(c)** Interactions between VH-FC ab8 and SARS-CoV-2 S produced in cells grown in the presence or absence of kifunensine. Experiments were done in triplicate; error bars indicate standard deviation (n = 3). Statistical significance was tested by two-way ANOVA with Sidak's post-test ($p > 0.05$ [ns, not significant], $p \leq 0.05$ [∗], $p \leq 0.01$ [∗∗], $p \leq 0.001$ [∗∗∗]).

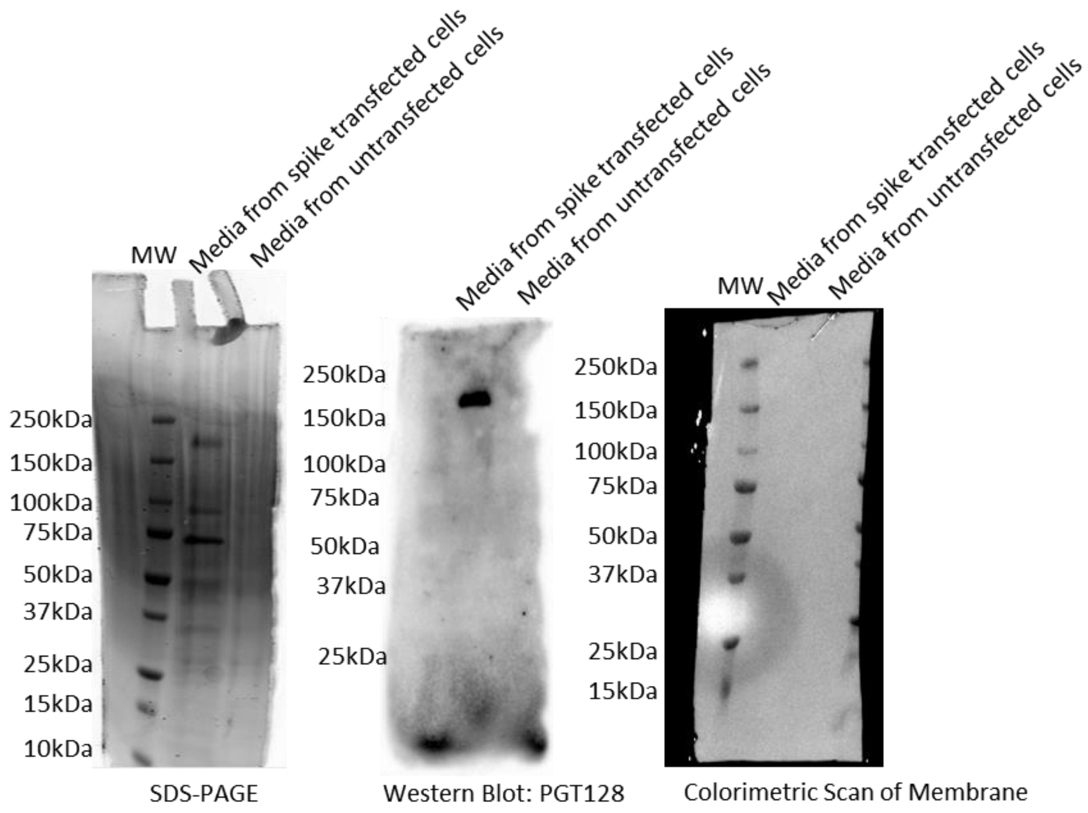

Figure S3. PGT128 specificity assessed via Western blot of unpurified SARS-CoV-2 Spike ectodomain in media. 10 microliters of unpurified media from untransfected cells or cells transfected with the SARS-CoV-2 soluble ectodomain were subjected to SDS-PAGE and Western blotting as described in the manuscript.

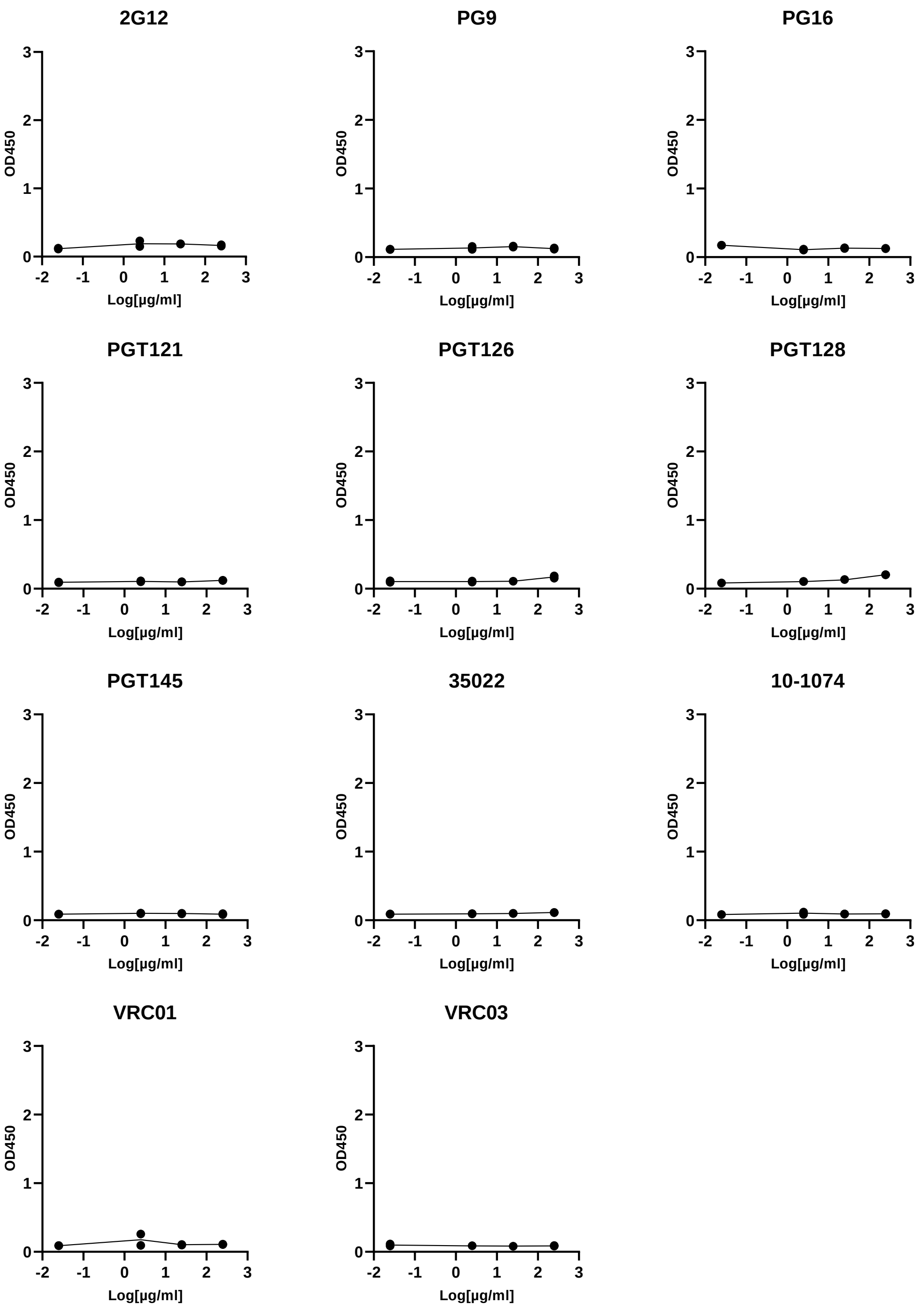

Figure S4. ELISA screen of glycan directed anti-gp120 antibody cross-reactivities to the SARS-CoV-2 Spike using the casein-based buffer. Serial dilutions of the indicated mAbs were assessed for SARS-CoV-2 S protein binding. VRC01 and VRC03 target the CD4 binding site within gp120 and are included here as negative controls. All ELISAs were performed using casein-based buffers (see methods). Experiments were done in duplicate and results are plotted as points.

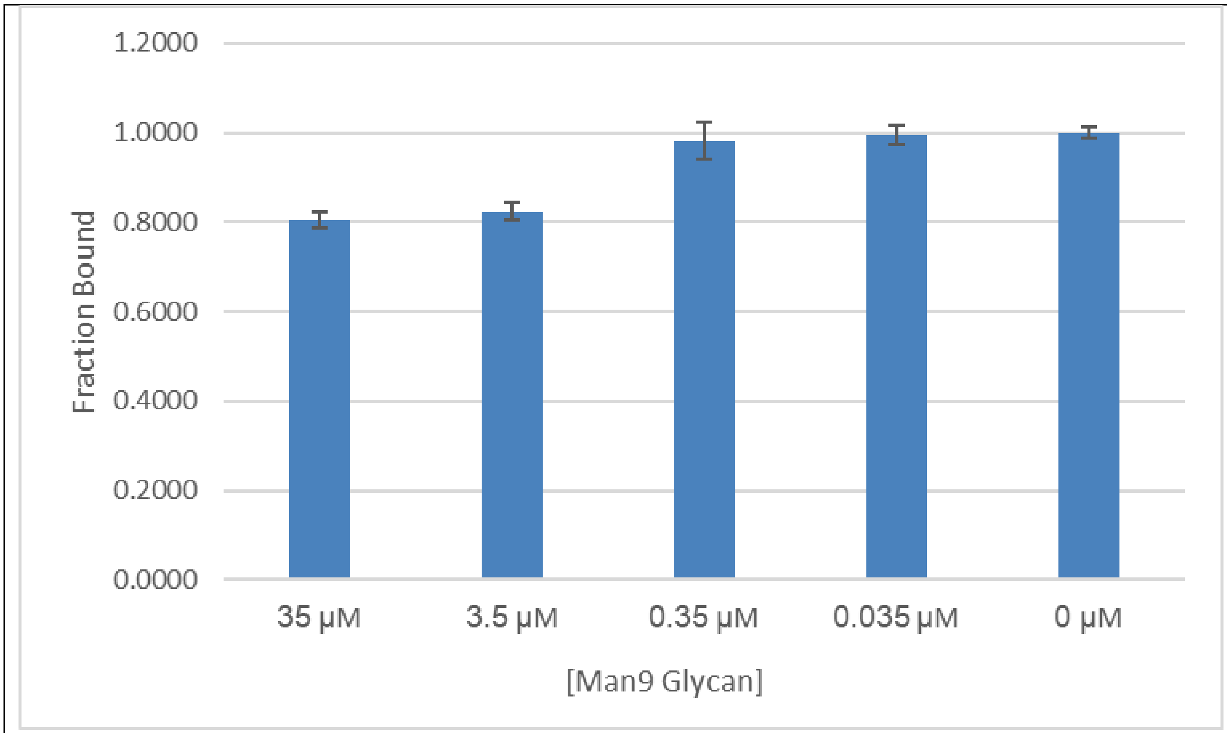

Figure S5. SARS-CoV-2 S ectodomain binding by anti-gp120 antibodies in the presence of Man9 glycan. Plates were coated with SARS-CoV-2 S ectodomain and incubated with dilutions of $(Man)_9(GlcNAc)_2$ along with a constant amount of PGT128. Antibody binding was quantified via ELISA. Experiments were done in triplicate; error bars indicate standard deviation (n = 3).

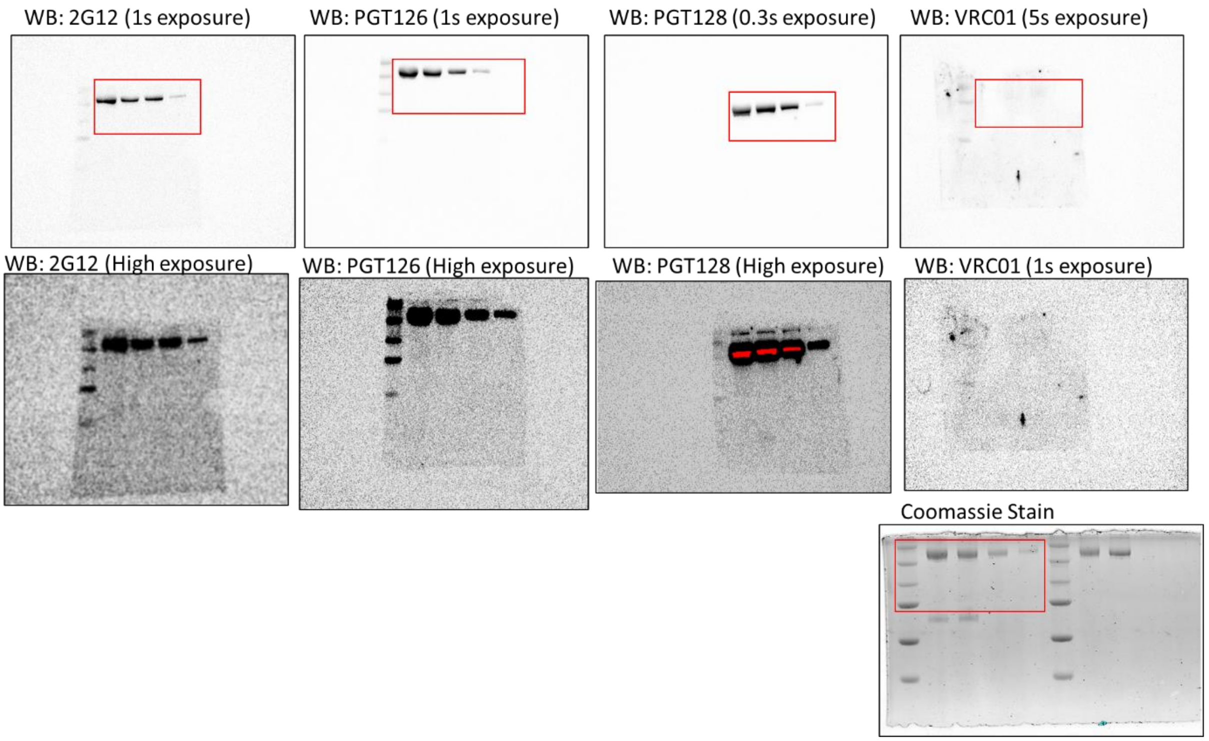

Figure S6. Uncropped gel and blot images related to Figure 3a. Cropped areas used in figure 3a are indicated with red boxes.

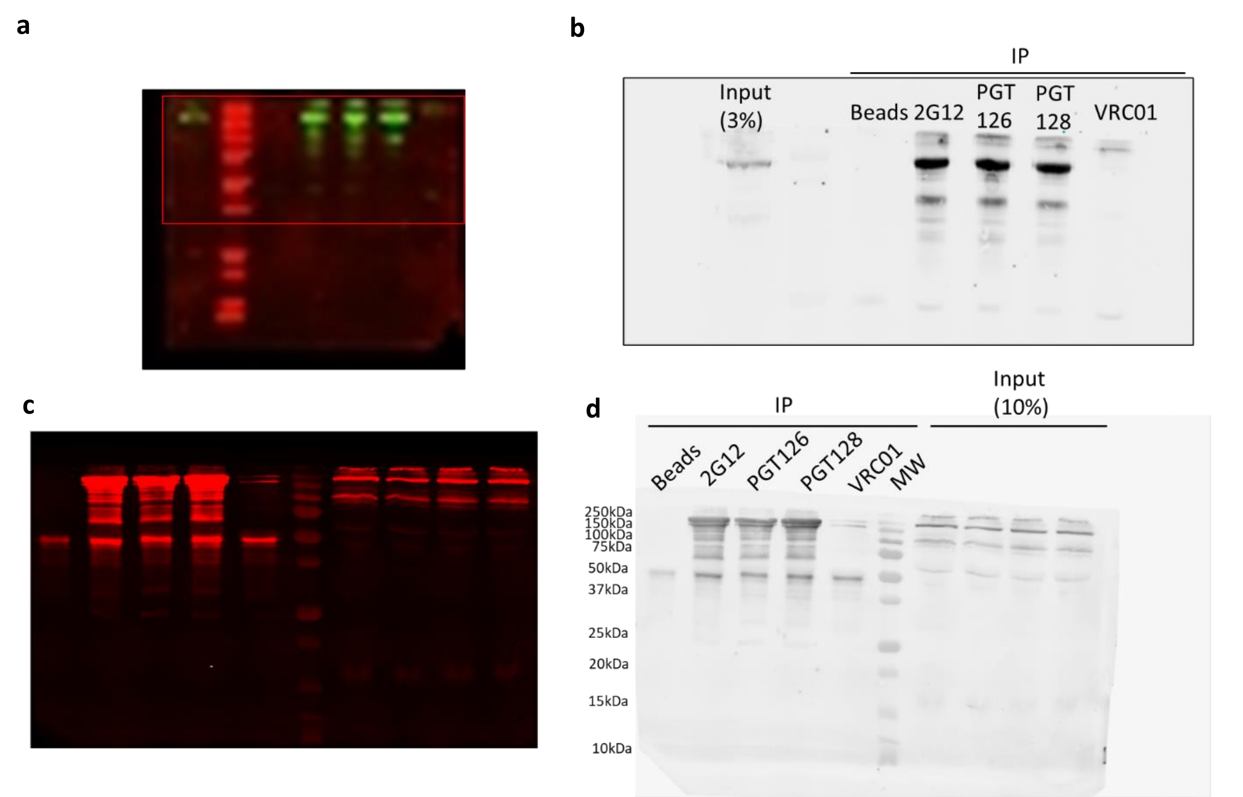

Figure S7. Uncropped blot images related to Figure 3b. (a) Raw multichannel image depicting ladder and antibody signals. The area indicated by the red box was scanned using single channel imaging and the uncropped image is presented in panel **(b)**. **(c)** and **(d)**: blots from a replicate pulldown experiment using an anti-strep tag antibody (Bio-Rad, cat#: MCA2489) for detection. **(c)** Raw single channel image depicting ladder and antibody signals. **(d)** annotated greyscale blot from **(c)**.

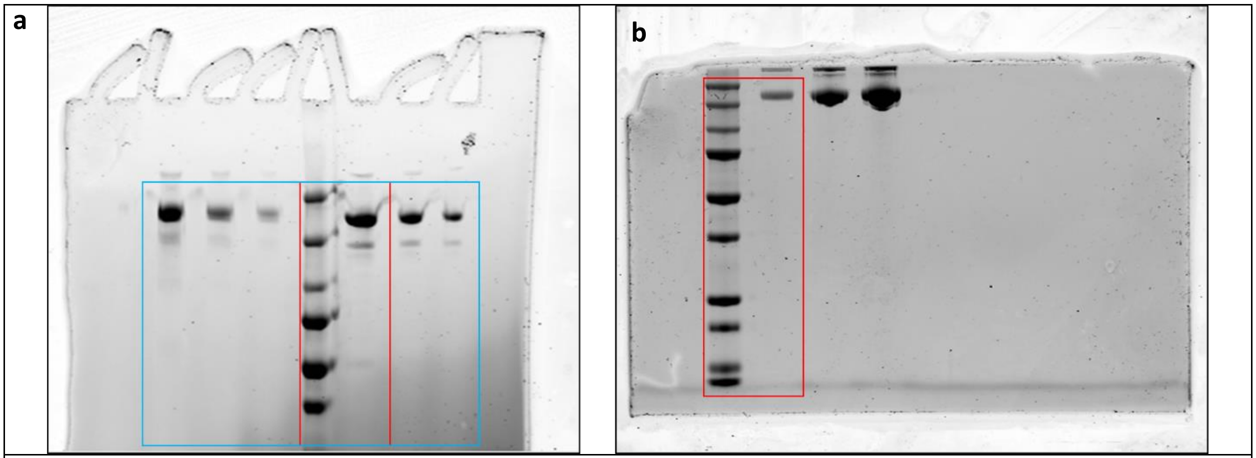

Figure S8. Uncropped gel and blot images related to Figure 4b and Figure S1. (a) Uncropped Coomassie stained SDS-PAGE gel related to figures 4b (cropped area outlined in blue) and figure S1 (cropped area outlined in red). **(b)** Uncropped Coomassie stained SDS-PAGE gel related to figure S1 (cropped area outlined in red).

References

1. Trkola, A. *et al.* Human monoclonal antibody 2G12 defines a distinctive neutralization epitope on the gp120 glycoprotein of human immunodeficiency virus type 1. *J. Virol.* **70**, 1100–1108 (1996).
2. Walker, L. M. *et al.* Broad neutralization coverage of HIV by multiple highly potent antibodies. *Nature* **477**, 466–470 (2011).
3. Julien, J. *et al.* Broadly Neutralizing Antibody PGT121 Allosterically Modulates CD4 Binding via Recognition of the HIV-1 gp120 V3 Base and Multiple Surrounding Glycans. **9**, (2013).
4. Garces, F. *et al.* Affinity Maturation of a Potent Family of HIV Antibodies Is Primarily Focused on Accommodating or Avoiding Glycans Article Affinity Maturation of a Potent Family of HIV Antibodies Is Primarily Focused on Accommodating or Avoiding Glycans. *Immunity* **43**, 1053–1063 (2015).
5. Pejchal, R. *et al.* A potent and broad neutralizing antibody recognizes and penetrates the HIV glycan shield. *Science* **334**, 1097–1103 (2011).
6. Lee, J. H. *et al.* Model Building and Refinement of a Natively Glycosylated HIV-1 Env Protein by High-Resolution Cryoelectron Microscopy Article Model Building and Refinement of a Natively Glycosylated HIV-1 Env Protein by High-Resolution Cryoelectron Microscopy. *Struct. Des.* **23**, 1943–1951 (2015).
7. Lee, J. H. *et al.* A Broadly Neutralizing Antibody Targets the Dynamic HIV Envelope Trimer Apex via a Long , Rigidified , and Anionic b -Hairpin Structure. *Immunity* **46**, 690–702 (2017).
8. Walker, L. M. *et al.* Broad and potent neutralizing antibodies from an African donor reveal a new HIV-1 vaccine target. *Science* **326**, 285–289 (2009).
9. Mclellan, J. S. *et al.* Structure of HIV-1 gp120 V1/V2 domain with broadly neutralizing antibody PG9. *Nature* (2011). doi:10.1038/nature10696
10. Pancera, M. *et al.* Structural basis for diverse N-glycan recognition by HIV-1 – neutralizing V1 – V2 – directed antibody PG16. *Nat. Publ. Gr.* **20**, (2013).
11. Pan, J., Peng, H., Chen, B. & Harrison, S. C. Cryo-EM Structure of Full-length HIV-1 Env Bound With the Fab of Antibody PG16. *J. Mol. Biol.* **432**, 1158–1168 (2020).
12. Shingai, M. *et al.* Antibody-mediated immunotherapy of macaques chronically infected with SHIV suppresses viraemia. *Nature* **503**, 277–280 (2013).
13. Gristick, H. B. *et al.* Natively glycosylated HIV-1 Env structure reveals new mode for antibody recognition of the CD4-binding site. *Nat. Struct. Mol. Biol.* **23**, 906–915 (2016).
14. Huang, J. *et al.* Broad and potent HIV-1 neutralization by a human antibody that binds the gp41-gp120 interface. *Nature* **515**, 138–142 (2014).
15. Wu, X. *et al.* Rational design of envelope identifies broadly neutralizing human monoclonal antibodies to HIV-1. *Science* **329**, 856–861 (2010).
